# Development of Folic Acid Functionalized Bilosomes for Delivery of Vorinostat to Breast Cancer Cells: Characterization and Cytocidal Effects on MCF-7 and 4T1 Breast Cancer Cell Lines

**DOI:** 10.34172/apb.43232

**Published:** 2024-12-14

**Authors:** Alireza Rashidi, Somayeh Taymouri, Mahboubeh Rostami, Mina Mirian

**Affiliations:** ^1^Department of Pharmaceutics, School of Pharmacy and Novel Drug Delivery Systems Research Centre, Isfahan University of Medical Sciences, Isfahan, Iran.; ^2^Department of Medicinal Chemistry, School of Pharmacy and Novel Drug Delivery Systems Research Centre, Isfahan University of Medical Sciences, Isfahan, Iran.; ^3^Department of Biotechnology, School of Pharmacy and Pharmaceutical Sciences, Isfahan University of Medical Sciences, Isfahan, Iran.

**Keywords:** Breast cancer, Bilosomes, Lithocholic acid, Vorinostat, Folate receptor, MTT assay

## Abstract

**Purpose:**

Breast cancer is a prevalent form of cancer in woman. Vorinostat (VOR) is a chemotherapeutic drug that has been utilized for treatment of breast cancer, but its effectiveness is limited due to low bioavailability and several side effects. The primary aim of this study is to prepare folic acid conjugated pegylated bilosomes containing VOR (FA-PEG- bilosomes) for the selective targeting of breast cancer cells with the FA receptor expression. Accordingly, lithocholic acid was used as a bile acid in bilosomes due to its anti-cancer and anti-proliferation effects.

**Methods:**

VOR loaded bilosomes were prepared using the thin-film hydration method and optimized by applying two-level fractional factorial design. Various properties of the considered bilosomes, including particle size, zeta potential, polydispersity index (PdI), encapsulation efficiency (EE) % and drug loading (DL) %, were then investigated and synthesized FA-PEG-Cholesterol was incorporated in the optimized bilosomes. The anti-cancer efficacy of VOR loaded FA-PEG- bilosomes was also evaluated *in vitro* using the MCF-7 and 4T1 breast cancer cell lines. Furthermore, drug free FA-PEG-bilosomes and bilosomes were evaluated for biocompatibility in the L929 cell line.

**Results:**

The optimized VOR loaded bilosomes exhibited spherical particles with the size of 305.33±18.50 nm, PdI of 0.37±0.03, zeta potential of -17.66±0.15 mV, EE of 92.91±0.22 % and DL% of 23.64±0.04%. Incorporation of FA-PEG-cholesterol in nanobilosomes increased the particle size and absolute value of zeta potential. *In vitro* cytotoxicity study also revealed that VOR loaded FA- PEG-bilosomes demonstrated a greater cytotoxic effect, as compared to the free VOR and VOR- bilosomes in both MCF-7 and 4T1 cancer cells.

**Conclusion:**

This showed that FA- PEG-bilosomes could be a promising formulation for the treatment of FA (+) tumors.

## Introduction

 Breast cancer (BC), the most prevalent malignancy, is considered treatable if detected at an early stage. However, if metastasis occurs, treatment difficulties and the fatality rates are increased rapidly due to the involvement of distant organs.^1^ Although chemotherapy plays an important role in cancer treatment, acute and long-term adverse effects on the patients’ healthy organs, as well as poor therapeutic effects caused by small molecules drugs which cannot effectively accumulate in tumor tissues, have limited clinical cure rates.^2^ Delivery of chemotherapeutic medications to tumor locations using nanomaterials has gained specific interest. Coupling of specific targeted molecules on the surface of nanoparticles (NPs) can improve the ability of NPs to penetrate into cells overexpressing a given receptor via receptor-mediated endocytosis and enhance the sustained release of an anticancer drug at the cellular targets to improve the efficiency of cancer chemotherapy.^3^ In addition, targeted NPs can bypass drug efflux via ATP-binding cassette transporters, e.g., P-glycoprotein (P-gp) and multi resistance-associated protein (MRP), which can help to overcome multi-drug resistance.^4^ Folic acid (FA) is a well-known vitamin capable of selectively binding with the folate receptor (FR), which is overexpressed in a variety of cancers, including liver, breast, ovarian and lung, while being minimally expressed in normal cells and tissues, thereby decreasing the potential of the side effects.^5^ Bilosomes are spherical-shaped vesicles in which bile salts are integrated into the membrane of liposomes and niosomes. Bile salts are endogenous detergents acting as a permeability enhancer in drug delivery, thereby facilitating the penetration of the drug through biological barriers. The inclusion of bile salts into the lipid bilayers of liposomes makes them repulsive to the intestinal bile salts in the gastrointestinal tract (GIT) and prevents the deformation of vesicle membrane and lysis, resulting in the premature release of the encapsulated molecules.^6^ Bilosomes have been utilized to improve the drug cytotoxic potential in the previous literature.^7,8^ Lithocholic acid (LCA) is a natural bile acid produced by gut microbes via biotransformation of primary bile acids (e.g. cholic acid and chenodeoxycholic acid) ^9^ and exert cytostatic effects on breast cancer cells through inducing mesenchymal-to-epithelial transition, increased antitumor immune response, OXPHOS and the TCA cycle.^10^ Therefore, in this study, we used it as bile acid in bilosomes. Despite the remarkable features of NPs, recognition of NPs as foreign entities and phagocytosis by macrophages may result in the rapid clearance of NPs and elimination before reaching tumor cells, thereby compromising drug delivery efficacy to tumor cells. Grafting a hydrophilic polymer onto the surfaces of NPs, such as poly ethylene glycol (PEG), can restrict the interactions between NPs and opsonin proteins that may mediate the phagocytic clearance, thereby resulting in the increased blood circulation half-life.^11^ According to this, PEGylated bilosomes offer more advantages over the non-PEGylated ones. Vorinostat (VOR) is the first histone deacetylase inhibitor approved by the US FDA for the treatment of cutaneous T-cell lymphoma (CTCL).^12^ Extensive preclinical/clinical investigations have also demonstrated the anti-cancer potential of VOR against other types of cancers, e.g., colon cancer, lung cancer, breast cancer and hepatocellular carcinoma, alone or combined with other cancer drugs. However, its use is accompanied by the gastrointestinal symptoms, hematologic abnormalities, taste disorders, pulmonary embolism and anemia.^12^ VOR has a low oral bioavailability (43%) because of the low aqueous solubility (0.2 mg/mL), low permeability and extensive first pass metabolism.^13^ Moreover, VOR has a short half-life in plasma due to rapid hepatic metabolism. Because of these properties, VOR is taken at a largely daily dose for the required therapeutic effect (400 mg by mouth once daily).^13^ Parenteral delivery of drug is also hindered due to the poor aqueous solubility.^14^ In the present study, we have utilized bilosomes as a drug delivery system to enhance the efficiency of VOR. VOR loaded bilosomes (VOR-bilosomes) were prepared by the film hydration method. In order to target the drug to the FR expressing cells, FA-PEG-Cholesterol conjugate was incorporated in optimized bilosomes; the antitumor activity of VOR loaded FA-bilosomes (FA-VOR-bilosomes) was then examined against MCF_7 _cells and 4T_1_ cells which overexpressed FA. The cellular uptake efficacies of FA-VOR-bilosomes in MCF7 cells were then evaluated by flow cytometry analyses and fluorescence microscopy.

## Materials and Methods

###  Materials

 VOR was purchased from Arasto pharmaceutical Chemicals Co, Iran. FA was purchased from Raha pharmaceutical Co, Iran. PEG 2000, coumarin 6 (C6), succinic anhydride, anhydrous dimethyl sulfoxide (DMSO), anhydrous dichloromethane, cholesterol, LCA, lecithin, 4 dimethylaminopyridine (DMAP), dialysis bag (molecular weight cut-off: 12000–14000 DA and 3000 DA), N, N’-Dicyclohexylcarbodiimide (DCC), 3-[4,5-dimethylthiazol-2-yl]-2,5-diphenyltetrazolium bromide (MTT) were purchased from Sigma Chemicals, USA. MCF-7 cells, 4T1 cells and L929 cells were purchased from Iranian Biological Research Center, Iran. Trypsin, fetal bovine serum (FBS), phosphate-buffer saline (PBS), Roswell Park Memorial Institute-1640 (RPMI-1640) medium, penicillin, and streptomycin were all purchased from Gibco Laboratories (USA). All other chemicals and solvents were of the analytical grade.

###  Synthesis of cholesterol succinate (CHS)

 A mixture of cholesterol (7.72 g, 20 mmol), succinic anhydride (3.02 g, 30 mmol), and DMAP (2.44 g, 20 mmol) was prepared in dichloromethane (100 mL). The excess amount of succinic anhydride was then utilized to ensure the complete reaction of cholesterol. The mixture was stirred at room temperature under N_2_ atmosphere overnight, and the progress of the reaction was monitored by TLC until no excess cholesterol was detected. After that, the reaction mixture was concentrated by rotary evaporation; the crude product was then recrystallized twice in acetic acid solution to obtain a white powder of CHS.^15^

###  Synthesis of PEGylated CHS (PEG -CHS)

 PEG- CHS was synthesized based on a previously reported procedure with a slight modification.^15^ A mixture of PEG (3.00 g, 1.5 mmol), CHS (0.49 g, 1 mmol), DMAP (0.125 g, 1 mmol), and DCC (0.42 g, 2 mmol) in 25 mL dichloromethane was then stirred under N_2_ atmosphere at room temperature for 24 h. After filtering the N, N-dicyclohexylurea (DCU) by-product, the filtrate was concentrated by rotary evaporation. Diethyl ether was then added to the residue and the white precipitate was collected. The purified product was obtained by silica gel column chromatography using chloroform/methanol eluent phase by gradually increasing methanol from 1 to 5%. A single spot of CHS-PEG was monitored by the TLC analysis (CHCl3: methanol, 9:1), using iodine vapor.

###  Synthesis of FA targeted PEGylated CHS (FA -PEG-CHS)

 At first, FA (500 mg, 1.13 mmol) was dissolved in DMSO (5 mL) with 1.5 equivalent amount of DCC and DMAP for at least 4 hours under N_2_ atmosphere. After that, the white precipitates of DCU were filtered and the filtrate was added dropwise to a solution of CHS-PEG (436 mg, 0.18 mmol) in DMSO (5 mL). The resulting solution was stirred for 24 hours at room temperature under the inert atmosphere. Finally, the resulting mixture was dialyzed (MW cut off, 30 000), respectively, against acetate buffer (pH of 5.5), phosphate buffer (pH of 7.8), and distilled water to remove the impurities. The purified powder of FA -PEG- CHS was then obtained after lyophilization.^16^

###  Experimental design

 A two-level fractional factorial design was used to optimize the independent factors and evaluate their effect on the characteristics of VOR- bilosomes. The lecithin content (A), lecithin (L) /LCA ratios (B), L/drug (D) ratios (C), and sonication time (D) were then considered as the independent variables at two levels, while the dependent variables were particle size, polydispersity index (PdI), zeta potential, encapsulation efficiency (EE)%, and drug loading (DL) % (Table 1). As a result, totally, 12 tests were designed in this study and each test was conducted 3 times (Table 2).

**Table 1:** Independent and dependent parameters studied for preparation of VOR- bilosomes

**Independent variables**	**Level 1**	**Level 2**	**Dependent variables**
A = Lecithin content (mg)	5	10	Particle size (nm)
B = Lecithin/Surfactant ratio	5	10	Polydispersity index (PdI)
C = Lecithin/Drug ratio	2.5	5	Encapsulation efficiency (%)
D = Sonication time (min)	2	4	Drug loading (%)
			Zeta potential (mV)

**Table 2:** Composition and physical properties of different VOR- bilosomes

**Formulations**	**Lipid content (mg)**	**Lipid/surfactant ratio**	**Lipid/drug ratio**	**Sonication time (min)**	**PdI**	**Zeta Potential** **(mV)**	**Particle size** **(nm)**	**EE %**	**DL %**
P100S10D2.5S4	100	10	2.5	4	0.40 ± 0.01	-18.73 ± 0.01	328.66 ± 12.58	84.83 ± 0.53	23.57 ± 0.11
P200S10D2.5S4	200	10	2.5	4	0.33 ± 0.02	-15.26 ± 0.30	327.33 ± 35.92	93.98 ± 0.22	25.47 ± 0.04
P100S10D5S2	100	10	5	2	0.40 ± 0.02	-17.50 ± 0.36	252 ± 34.17	73.97 ± 0.47	11.85 ± 0.06
P100S5D2.5S2	100	5	2.5	2	0.40 ± 0.05	-16.16 ± 0.25	327.66 ± 3.51	84.94 ± 0.72	22.06 ± 0.14
P100S10D5S4	100	10	5	4	0.38 ± 0.02	-15.13 ± 0.15	255 ± 43.71	76.91 ± 0.98	12.26 ± 0.13
P200S5D5S4	200	5	5	4	0.39 ± 0.04	-15.26 ± 0.30	308.33 ± 35	87.10 ± 0.30	12.67 ± 0.03
P100S5D2.5S4	100	5	2.5	4	0.41 ± 0.02	-15.80 ± 0.26	270.8 ± 46.95	86.60 ± 0.49	22.40 ± 0.09
**P200S5D2.5S4**	**200**	**5**	**2.5**	**4**	**0.37±0.027**	**-17.66±0.15**	**305.33±18.50**	**92.91±0.22**	**23.64±0.04**
P200S10D2.5S2	200	10	2.5	2	0.36 ± 0.04	-17.53 ± 0.45	380.66 ± 25.14	93.54 ± 0.34	25.38 ± 0.07
P100S5D5S2	100	5	5	2	0.43 ± 0.04	-16.83 ± 0.25	296.33 ± 23.43	77.90 ± 1.05	11.49 ± 0.13
P200S5D5S2	200	5	5	2	0.35 ± 0.04	-16.36 ± 0.32	205.33 ± 19.85	87.25 ± 0.41	12.69 ± 0.05
P200S10D5S2	200	10	5	2	0.39 ± 0.02	-17.06 ± 0.47	241.93 ± 37.30	85.13 ± 0.80	13.40 ± 0.11

The bold line stands for The optimized VOR loaded bilosomes.

###  Preparation of VOR- bilosomes

 VOR-bilosomes were prepared using the thin-film hydration method with a modified literature procedure; this was followed by probe sonication.^17^ For this, accurately weighted amounts of VOR (40-80), lecithin (100-200), and LCA (20-40) were dissolved in 10 ml of a methanol/chloroform mixture (2:1 respectively). The solvent was then evaporated under vacuum using a rotary evaporator rotating at 280 rpm to form a thin solid film on the inner walls of the round-bottomed flask. The formed thin layer film was then hydrated by adding 10 cc deionized water. The dispersion was then sonicated at 40 W with a probe sonicator (bandelin electronic, Germany) for 2-4 minutes at periodic 2 sec pulse-on time and 2 sec pulse-off time. A two-level fractional factorial design was then used to optimize the independent factors and evaluate their effect on the characteristics of VOR- bilosomes (Table 1). As a result, totally, 12 tests were designed in this study and each test was conducted 3 times (Table 2). To prepare FA modified long circulating bilosomes (FA-VOR-bilosomes), 10 (w/w) % of lecithin in the optimized formulation was replaced with 5 (w/w) % PEG -CHS and 5 (w/w) % FA -PEG- CHS. Blank bilosomes and FA- bilosomes were prepared in same manner, except that no drug was used in the formulation.

###  Characterization VOR- bilosomes

####  Mean particle size, PdI and zeta potential

 The mean particle size, PdI, and zeta potential were measured after 10-fold dilution with distilled water using the dynamic light scattering (DLS) technique at 25 °C with a zeta sizer (ZEN 3600 Malvern, UK)

####  DL% and EE% determination

 EE of the VOR in the bilosomes was determined using a modified literature procedure.^18^ For this, 0.5 mL of each formulation was centrifuged (Microcentrifuge 5430 Hamburg, Germany (at 12 000 rpm for 15 minutes in Amicon microcentrifuge tubes (cutoff 10 000 Da, Ireland). The filtrate containing the free drug was diluted tenfold with distilled water and the amount of un-encapsulated drug was then determined spectrophotometrically at 262.5 nm using a UV-VIS spectrophotometer (Shimadzu®, Japan). Finally, the following equations were used to determine EE% and DL%:


Eq. 1 $$EE\%=\left(\frac{total\;amount\;of\;drug\;added-free\;drug}{total\;amount\;of\;drug\;added}\right)\times 100$$

Eq. 2 $$DL\%=\frac{loaded\;drug}{\left(loaded\;drug+polymer\;content\;\right)}\times 100$$

To eliminate the interference from other formulation components, the same procedure was done for drug free NPs as blank.

###  Drug release studies

 0.5 mL of the optimized and targeted formulation was put in a dialysis bag (molecular cut off 12 000 Da). The dialysis bag was then placed in 100 mL of the phosphate buffer solution (PBS, pH = 7.4) to provide the sink condition. The release medium was stirred continuously at 360 rpm by a magnetic stirrer and temperature was kept at 37 ˚C. At a specified time, 1 mL of release medium was removed and replaced with an equivalent fresh PBS. Finally, the released VOR was measured using a UV spectrophotometer at 262.5 nm. The solution obtained from a dialysis test was carried out using unloaded NPs to serve as the control.

###  Scanning electron microscopy (SEM)

 To characterize the shape of FA-VOR-bilosomes, a drop of formulation was deposited onto a carbon coated copper grid and allowed to dry in air. The sample was then coated with gold and examined using SEM operating at the voltage of 10 kV.

###  Stability of FA-VOR- bilosomes in the simulated gastric fluid (SGF)

 The stability of the optimized FA-VOR-bilosomes was investigated in simulated gastric fluid (SGF). For this purpose, 1 mL of formulation was added to 9 ml of SGF and the mixture was incubated at 37 °C for 2 hours.^19^ After incubation, the bilosomes were checked for particle size, PdI, zeta potential and EE%, as described above.

###  Ex vivo intestinal permeation studies

 Ex vivo intestinal permeation of VOR from its suspension and FA-bilosomes was evaluated using the non-everted gut sac technique, according to method reported in a previous study.^20^ Male Wistar rats weighing 180-220 g were then used for the study. For this, the animals were anaesthetized using 75 mg/kg ketamine and 10 mg/kg xylazine. Following midline laparotomy, the upper end of the duodenum and the lower end of the ileum were cut to remove the small intestine; then 8 cm long segment of jejunum having a diameter of 3 mm was separated and washed with cold Kreb’s Ringer phosphate buffer (KRPB) for the removal of the food residue. Finally, 1 mL of FA-VOR-bilosomes or VOR suspension was filled using a blunt needle in one end of the ligated sac; then the other end of the sac was tied. Each sac was placed in 25 mL of the KRPB buffer saline (pH 7.4), maintained at 37 °C, stirred at 360 rpm using a magnetic stirrer, and constantly aerated with oxygen (10–15 bubble/min). At specified times, all of the buffer was withdrawn and replaced with 25 mL of the fresh buffer. The solutions were then freeze-dried and the resulting powder was dissolved in ethanol. The solution was centrifuged (5 minutes, 2500 rpm) and the absorbance of the supernatant was measured using a UV spectrophotometer at 262.5 nm. The cumulative amount released per unit area of sac (μg/cm^2^) in the receptor compartment was then calculated.

###  Cell culture

 The MCF-7, 4T1 and L929 cells were cultured in the RPMI-1640 medium supplemented with 10% (v/v) FBS, 100 IU/mL penicillin, and 100 µg/ml streptomycin sulfate under the standard condition of 5% CO_2_ and 95% humidity at 37 °C.

###  In vitro cell cytotoxicity study

 The anti-proliferation activity of the free VOR, optimized VOR- bilosomes, and FA-VOR-bilosomes on MCF_7 _and 4T_1_ cells was evaluated using the MTT assay. For this purpose, 180 μL of the cell suspension containing 5 × 10^4^ cells/ml was seeded in a 96-well plate and incubated at 37 °C for 24 hours. The cells were then exposed to 20 µL of each formulation with different VOR concentrations ranging from 2 to 36 µM for 48 h. After the incubation period, the medium was removed and 20 µl of MTT solution (5 mg/mL in PBS) was added to each well and incubated for another 3 h at 37 °C. Subsequently, the medium was removed, and 150 µL of DMSO was added to each well to dissolve the formazan precipitate. Finally, the absorption was measured at 570 nm using the microplate reader (BioTek, USA), and the cell viability % was determined by applying the following equation:


Eq. 3 $$Cell\;viability\%=\left(\frac{mean\;absorbance\;of\;each\;group-mean\;absorbance\;of\;blank}{mean\;absorbance\;of\;negative\;control-mean\;absorbance\;of\;blank}\right)\times 100$$

The negative control and blank are represented by the non-treated cells and the culture medium, respectively.

 The cytotoxic effects of drug-free bilosomes and drug-free FA targeted bilosomes against MCF-7, 4T1 and L929 cells were then determined utilizing the methodology detailed above in concentrations equivalent to the amount of the materials used in each concentration of the drug-loaded formulations.

###  Cellular uptake study

 The impact of conjugated FA-PEG-CHS on the bilosomes uptake was investigated using flow cytometry and fluorescence microscopy studies. For this, the optimized bilosomes and FA targeted bilosomes were loaded with 1 mg C6 as a florescent probe instead of VOR. MCF-7 cells (10^5^ per well) were then seeded in 12 well plates, incubated for 24 h and treated with 200 ng/mL of C6 loaded FA - bilosomes and the optimized bilosomes for 1 and 2 h. At the end of each incubation time, the cells were washed three times with PBS and the cellular uptake was observed using fluorescence microscopy (CETI, Belgium).

 To quantitatively study the cellular uptake, the MCF-7 cells were incubated with optimized bilosomes and FA targeted bilosomes for 2 hours. After incubation, the cells were washed three times with PBS, harvested by trypsinization, collected by centrifugation and finally, resuspended in 1 mL of PBS for flow cytometric analysis (BD FACSCalibur, US).

## Results and Discussion

###  Synthesis and characterization of CHS

**Figure 1:**
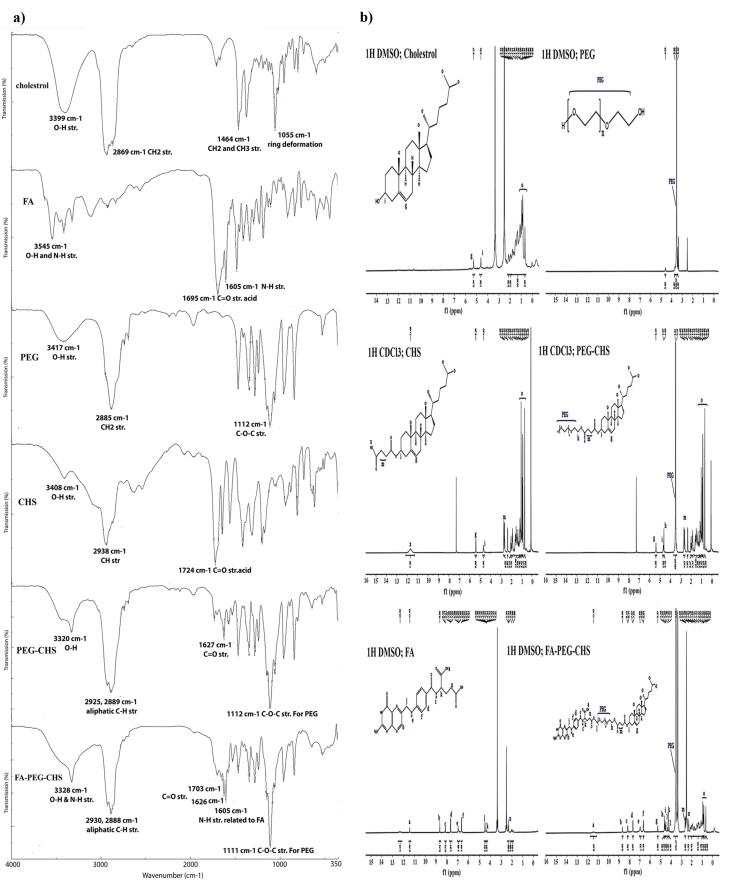
a) FTIR spectra and b) 1HNMR spectra of cholesterol, FA, PEG2000, CHS, PEG-CHS, and FA-PEG-CHS

 The confirmation of the structure of CHS was done using 1H NMR and FTIR spectra. As shown in Figure.1a, in the FTIR spectrum of CHS, the absorption bands of cholesterol were visible in the related areas. The O-H stretch of cholesterol at 3399 cm^-1^ could be seen at the absorption band at 3408 cm^-1^ in CHS. Similarly, the CH_2_ stretch of cholesterol at 2869 cm^-1^ was visible in CHS at 2938 cm^-1^. Additionally, the C = O stretch of CHS at 1724 cm^-1^ confirmed the formation of this compound. Figure 1b displays the HNMR spectra of cholesterol and CHS. In the spectrum of CHS, the cholesterol signals appeared in the correct areas. The signals related to protons attached to oxygen could be observed at 4.65 ppm (Hi), and the signals around 5.27 ppm were associated with the protons of the double bond group (Hg). The cholesterol moiety signals (Ho) correctly appeared around 0.69-0.92 ppm, and the succinate part (Hm) emerged around 2.62-2.69 ppm.

###  Synthesis and characterization of PEG-CHS and FA-PEG-CHS

 The structure of PEG-CHS and FA-PEG-CHS was confirmed by 1H NMR and FTIR spectra. Figure.1a presents the FTIR spectra of cholesterol, FA, PEG, CHS, CHS-PEG, and CHS-PEG-FA. In the FTIR spectrum of CHS-PEG, the absorption bands at 3320 cm^-1^ (O-H str. for PEG), 2925 cm^-1^ and 2889 cm^-1^ (aliphatic C-H str. for CHS and PEG), 1627 cm^-1^ (C = O str. for CHS) and 1112 cm^-1^ (C-O-C str. for PEG) confirmed the formation of this compound. Meanwhile, in the FTIR spectrum of CHS-PEG-FA, the absorption bands at 3328 cm^-1^ (O-H & N-H str. for FA), 2930 cm^-1^, 2888 cm^-1^ (aliphatic C-H str. for CHS, PEG and FA), 1703 cm^-1^, 1626 cm^-1^ (C = O str. for CHS and FA), 1605 cm^-1^ (N-H str. for FA), and 1111 cm^-1^ (C-O-C str. for PEG) confirmed the formation of this compound from its ingredients. Also the clear differences in the overall characteristics of the spectrum in comparison to its ingredients are the convincing proofs. When comparing CHS-PEG-FA and CHS-PEG, the presence of N-H str related to FA at 1605 cm^-1^ indicated the presence of FA, thus highlighting the difference between these two substances. The final proofs were retrieved from the 1HNMR spectra. The 1HNMR of the final product has been presented in Figure. 1b. in comparison to its ingredients. As it is clear from the final product spectrum, the characteristic signals of CHS, FA, and PEG were correctly appeared in the related area with some shifts for the protons near the reaction site. CHS signals appeared around 0.69-0.92 (Ho, cholesterol moiety), 2.62-2.69 ppm (Hm, succinate part), 4.65 ppm (Hi), and 5.27 ppm (Hg). FA characteristic peaks were around 2.38 ppm (Hn), 4.35 ppm (Hk), 4.5 ppm (Hj), and those which correctly appeared above 6.5 ppm. Also, PEG characteristic signals appeared at 3.52 ppm (PEG, protons of backbone), 4.22 ppm (Hl), and 4.70 ppm (Hh). It should be noted that signals related to FA (-CH_2_, Hn) and PEG (-CH_2_, Hl) experienced significant changes after the final reaction of FA and CHS-PEG, which demonstrated the correct chemical conjugation. FA characteristic signal assigned as H_n_ was moved from 2.32 to 2.28 ppm and protons assigned as H_l, _which belonged to PEG, appeared around 3.5 ppm in the CHS-PEG conjugate, which moved to 4.22 ppm after conjugation via the ester bond to FA.

###  Characteristics of VOR- bilosomes

**Figure 2:**
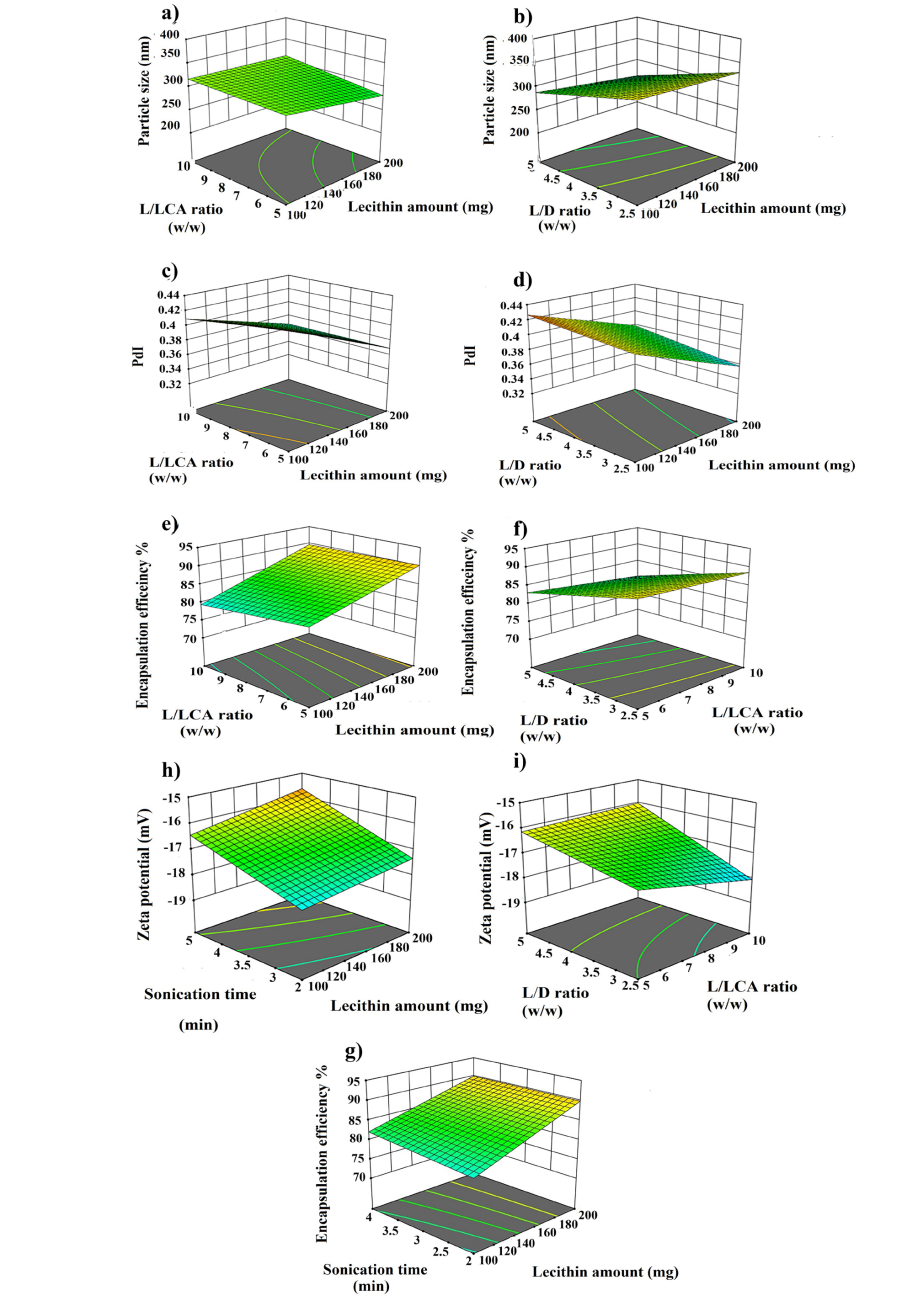
3D response surface plots showed the impacts of the predetermined factors on particle size, PdI, EE% DL% and zeta potential

**Figure 3:**
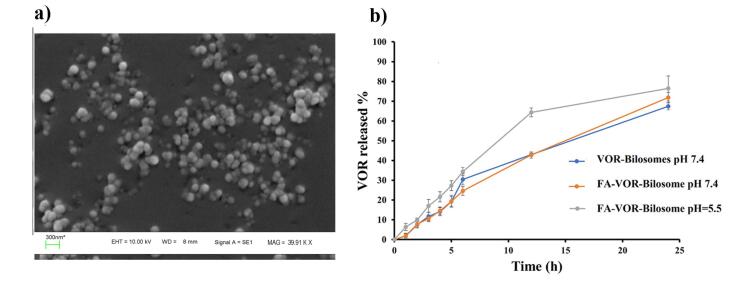
a) SEM image of optimized FA-VOR-bilosomes b) VOR release profile from the optimized bilosomes at pH = 7.4 and FA -bilosomes at pH = 7.4 and pH = 5.5

 VOR- bilosomes were prepared by the thin-film hydration method. Finding the right combination of process and formulation variables that can lead to the production of high quality products is the biggest challenge in the development of pharmaceutical products. Statistical experimental designs provide the possibility of studying the impacts of variables and their interaction, with fewer experiments and less time. In the present study, the fractional factorial design was used to optimize and evaluate the effects of four different parameters on the VOR- bilosomes. The experimental results of the physicochemical characterization of VOR- bilosomes are summarized in Table 2. 3D response surface plots were then used to graphically examine the impacts of the predetermined factors on the evaluated responses, as shown in Figures 2 and 3. The results of each response were fitted into 2 factor interaction model and the effect was also presented by a mathematical polynomial equation. In this equation, the positive sign shows the increase in the respective response parameter with raising the level of one variable, while the negative one indicates the decrease in the response parameter with increasing the level of one variable.

###  Effect of formulation variables on particle size 

 Particle size and size distribution are important factors for nanoscaled formulation as they have impact on the drug’s release rate, stability, cellular uptake, bio-distribution and therapeutic effect of the drug.^21^ As displayed in Table 2, the VOR- bilosomes formulations were in the range of 205 to 380 nm. According to the ANOVA results, lecithin amount (A), L/LCA ratios (B), L/D ratios (C), AB, AD, BC, BD and CD exhibited a significant impact on the particle size. Equation 4 and Figure 2 show the effect of the independent variables on the particle size.


Eq. 4 Particle size=+305.49−8.48 A+9.02 B−29.83 C+5.90 AB−3.84AC+12.94 AD−23.01 BC−12.06 BD+27.03 CD+14.60 ABC

From equation 4, it could be seen that lecithin and L/D ratios had a negative effect, while L/LCA ratios (B) exhibited a positive effect on particle size. As the lecithin amount was raised from a low one to a high one, the particle size was decreased (Figure 2a, *P* < 0.05). This could be due to the reduction of surface free energy resulting from high hydrophobicity, which led to a larger number of particles that were smaller in size. This was also in accordance with the literature.^22^ The particle size was also decreased by increasing the L/D ratio (Figure 2b, *P* < 0.05). This could be due to the hydrophobic nature of VOR. Packing of these molecules in the bilayer membrane hindered the compact packing of lipid vesicles, which might increase the vesicle size.

 On the other hand, particle size was increased when raising the L/LCA ratio, which was accompanied with the decrease of the LCA content (Figure 2a). LCA is a non-ionic surfactant with the HLB value of about 2.5.^23^ The increase in the surfactant concentration reduced the interfacial tension between lipid and aqueous phase, resulting in the smaller particle size. In addition, solubilization of the surfactant within the lipid bilayer owing to hydrogen bonding between the alkyl chain of the surfactant and the polar head of phospholipid might also cause the flexibility of bilosome and consequently, vesicle size reduction. This finding was also in agreement with the studies previously conducted by other researchers.^22-24^ Particle size is expected to decrease with the increase of the sonication time. However, contrary to this, no significant change in the size of vesicles was observed with increasing the sonication time.

###  PdI

 PdI is an index used to describe the variation of particle size in a population of particles. As shown in Table 2, the PdI value ranged from 0.33 to 0.43, which was less than 0.5, showing a relative uniform size distribution.^25^ The following equation shows the relationship between PdI and the independent variables.


Eq. 5 PdI=+0.3944−0.0257 A−0.0059 B+0.0086 C+0.0049 AB+0.0020 AC−0.0061 BC−0.0122 BD+0.0047 CD+0.0103 ABC

ANOVA analysis showed that lecithin amount (A), L/LCA ratios (B), L/D ratios (C), AB, BC, and BD exhibited a significant impact on PdI. According to Figure.2c and Equation 5, it was found that as the amount of lipid and L/LCA ratios were increased, PdI was decreased and more uniform particles were formed. In contrast, for the L/D ratios, as the ratio was increased, the particles became less uniform and the PdI value was increased (Figure 2d).

###  EE %

 The obtained value for EE % was in the range of 73% - 93 %. Such a high EE% was due to the hydrophobic nature of both VOR and carrier, which helped VOR to easily get impregnated in the hydrophobic backbone of bilosomes.^26^ The ANOVA results also showed that the amounts of lecithin (A), L/LCA ratios (B), L/D ratios (C), sonication time (C), AB, AD and BC had a significant impact on EE%. Equation 6 and Figure 2e-2f represent the influence of the independent factor on EE %.


Eq. 6 EE=+85.43+4.5 A−0.8908 B−3.65 C+0.6108 D+0.5809 AB−0.5877 AD−0.4344 BC+0.2347 BD

According to equation 6, lecithin amount and sonication time showed a positive effect. Meanwhile, lecithin/LCA and lecithin/D ratio had a negative impact on EE %. The decrease of L/LCA ratio, i.e., the rise of LCA concentration increased EE% (Figure 2e). Increasing the concentration of LCA resulted in the rise of EE %, since LCA could act as a solubilizing and destabilizing agent in the bilayer, thereby increasing the flexibility of bilosomes and solubility of VOR into the lipid bilayer.^24,27^ Moreover, increasing the lecithin amount in the formulation resulted in the enhancement of VOR EE % (Figure 2e), which could be attributed to raising the viscosity of the organic phase solution that prevented the leaching of VOR in the aqueous phase. In addition, higher phospholipid content provides more spare space to accommodate the excessive drug.^28^ A similar finding has been reported for quercetin loaded chitosan coated bilosomes.^29^ As shown in Figure 2f, as L/D ratio was declined, EE % was increased. It was possibly induced by the higher VOR concentration, as more of the drug would be available for entrapment into vesicles. Additionally, it showed that saturation concentration in particles was not achieved and they could contain more drug.^30^ Meanwhile, sonication time had a positive effect on EE%, such that the increase in sonication time raised EE%, which was in line with a previous study (Figure 2g). The higher sonication may increase the drug solubility in the lipid matrix, resulting in the rise of EE%.^31^

###  DL%

 The DL% value varied from 11 % to 26%. The results of the experimental design showed that lecithin amount (A), lecithin/LCA ratio (B), lecithin/D ratio (C), sonication time (C), AB, AC, AD and BC had a significant impact on DL%.


Eq. 7 DL=+18.07+0.7367 A+0.5087 B−5.60 C+0.1022 D+0.1241 AB−0.1331 AC−0.0851 AD−0.2175 BC+0.0234 BD−0.0362 ABC

As illustrated in equation 7, a strong positive correlation was found between lecithin amount, L/LCA ratios and sonication time, while DL% was negatively affected by the L/D ratio. Except for the L/LCA ratios, DL% changed with these variables along with the change in the entrapped drug, as reported in the previous section; so the higher the entrapped drug, the higher DL%.

###  Zeta potential

 Zeta potential, which represents the surface charge of particles, is an important parameter determining the magnitude of the electrostatic repulsion or attraction between particles in a liquid suspension; it is used for describing the stability of a dispersion system.^32^ The zeta potential of VOR- bilosomes was between -15 and -18.73 mV. As LCA is a non-ionic bile salt, the negative value of the zeta potential resulted from negatively charge phospholipid in lecithin, and this was in line with the results previously reported.^33^

 According to the ANOVA results, lecithin amount, L/LCA ratios, L /D ratio, sonication time, AB, BD and BC had a significant impact on the zeta potential. According to Equation 8, except for the L/LCA ratio, which had a positive effect, lecithin amount (A), L /D ratio (C) and sonication time (C) had a negative influence on the net value of the zeta potential.


Eq. 8 Zeta Potential=−16.80+0.3125 A−0.2333 B+0.7250 C+0.7625 D+0.6625 AB+0.0833 AD+0.2917 BC+0.3958 BD+0.1042 CD−0.6708 ABC

Lecithin is a zwitterionic type phospholipid in water that possesses negatively charged phosphoric and positively charged N^+^ groups. The decrease in the magnitude of the zeta potential with increasing the lecithin concentration may be due to the crystalline reorientation of lipid; as such, more choline moieties present on the exterior part of bilosomes and phosphatidyl groups could hide themselves in the molecule back (Figure 2h).^34^ The greater amount of LCA also made the zeta potential less negative (Figure 2i), since LCA has a non-ionic property and the lower concentration of phospholipid in the case of decreased L/LCA ratios reduced the zeta potential.^34^ In contrast, we observed an increase in the zeta potential value when the drug concentration was raised (Figure 2i), which could be attributed to the accumulation of more hydroxyl and carboxyl groups of drug molecules on the surface of particles owing to the greater entrapment of the drug at the lower L/D ratios.^35^

###  Optimization

 The Design Expert software was used to select the best formulation. The criteria for this selection included a smallest particle size, the lowest PdI, and the highest absolute value of zeta potential and EE%. After a comprehensive analysis, the formulation P200S5D2.5S4 was chosen. This formulation was prepared using 200 mg of lecithin, 40 mg of LCA, and 80 mg of the drug. The sonication time was set to 4 minutes. The formulation met the criteria for the optimal performance with the desirability of 63%, and it was one of our previous formulations too. Meanwhile, the particle size of the optimized formulation was 305.33 ± 18.50 nm, PdI was 0.37 ± 0.03, DL was 23.64 ± 0.04%, EE was 92.91 ± 0.22 % and the zeta potential was -17.66 ± 0.15 mV. In the continuation of our work, FA-VOR-bilosomes were prepared by replacing 10 (w/w) % of lecithin in the optimized formulation with 5 (w/w) % PEG - CHS and 5 (w/w) % FA -PEG-CHS. The physicochemical properties of FA-VOR-bilosomes are summarized in Table 3. The particle size of FA-VOR-bilosomes (321.43 ± 15.46) was increased, as compared to the non-targeted ones, due to the bulkiness of the FA moiety.^36^ Additionally, the zeta potential of FA-VOR-bilosomes (-20.56 ± 0.92) was slightly more negative, as compared to the non-targeted ones, which was owing to the presence of the negatively charged residues of the FA moiety on the surface of bilosomes.^36^ The stability of FA-VOR-bilosomes after 2 hours incubation of this formulation in the simulated gastric condition was also evaluated. The particle size, zeta potential, PdI and EE% were 335.29 ± 13.03, 0.41 ± 0.05, -18.06 ± 0.54 and 92.10 ± 0.22, respectively, which were comparable to those measured before the experiment. This showed that the FA-VOR-bilosomes kept their integrity and drug-retention properties, possibly due to the protective effect of PEG on the surface of the bilosomes against pH variations.^37^ VOR release profiles from the optimized bilosomes at pH = 7.4 and from FA-bilosomes at pH = 7.4 and pH = 5.5 are shown in Figure 3b. As can be seen, the release profile of the drug from FA-VOR-bilosomes was similar to that of optimized bilosomes. The release of VOR from FA-VOR-bilosomes was increased in acidic pH, as compared to physiological pH. For instance, the amounts of released VOR at the sampling times of 12 and 24 hours were 42.78% and 71.86% at pH 7.4, respectively. Correspondingly, the amounts were 64.34% and 76.43% at pH = 5.5. The slightly increased drug release from FA-VOR-bilosomes in acidic pH, as compared to physiological pH, was due to the protonation of the amine moiety of VOR, leading to the increased drug solubility.^38^ This pH-dependent release behavior could be beneficial for targeted drug delivery to the slightly acidic tumor microenvironment. Further, the SEM image (Figure 3b) clearly revealed that the optimized FA-VOR-bilosomes had spherical shapes lower in size when compared to those obtained by the DLS method. This is since, in the DLS measurements, the hydrodynamic layer on the surface of NPs was considered. Meanwhile, in the SEM analysis, particles were in the dry state.

**Table 3:** Physical properties of optimized FA-VOR-bilosomes

**Formulations**	**Particle size (nm)**	**PdI**	**Zeta Potential (mV)**	**EE %**	**DL %**
FA-VOR-bilosomes	321.43 ± 15.46	0.39 ± 0.02	-20.56 ± 0.92	94.24 ± 0.61	18.83 ± 1.67

###  Ex vivo permeation studies

**Figure 4:**
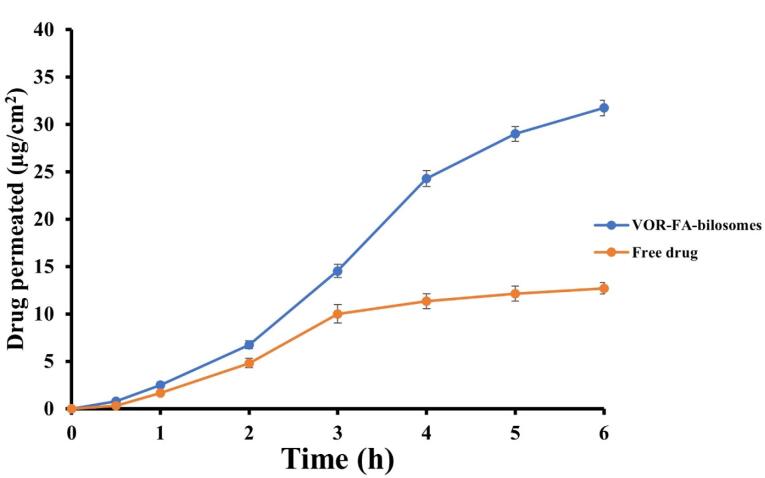
Intestinal permeation profiles of VOR- FA-bilosomes and free drug

Figure 4 shows the intestinal permeation profiles of the VOR- FA-bilosome and drug suspension. As can be seen, the cumulative amount of VOR permeated after 6 hours for VOR- FA-bilosome was higher than that of the drug suspension. This increase in permeability could be attributed to the small size of the bilosomal carriers and the successful entrapment of VOR into these carriers, which caused the faster diffusion of the drug across the intestinal membrane. These were consistent with other results concerning the enhanced drug delivery via bilosomes.^6,39^ Furthermore, FA-modification could facilitate the interaction of bilosomes with enterocytes and enhance the transcytosis of bilosomes, since FA receptors are abundantly expressed at the apical (luminal) surface of the intestinal cells.^40^

###  Cellular uptake study

**Figure 5:**
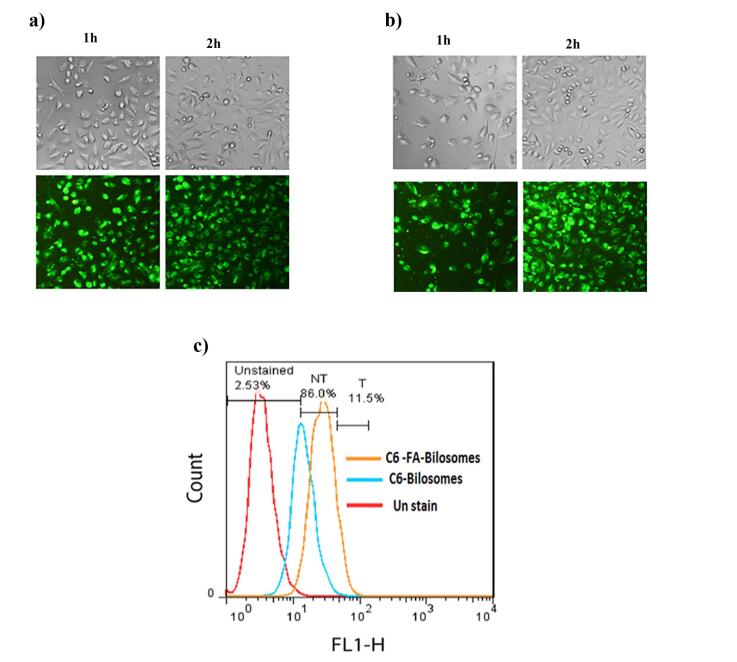
The visible light photos and fluorescent light photos of MCF-7 cells after 1 and 2 h incubation at 37 °C with a) C6 loaded non-targeted bilosomes and b) C6 loaded FA-bilosomes c) Flow cytometry profiles of MCF_7_ cells after 2 h incubation with culture media (red), non-targeted bilosomes (blue) and C6 loaded FA-bilosomes (orange)

 C6 loaded FA-bilosomes and non-targeted bilosomes were used to trace cellular uptake and investigate whether the conjugation of FA facilitated the internalization of FA-bilosome in the MCF_7_ cells to overexpress the FA receptor. The cellular uptake study was performed visually by florescent microscopy and quantitatively using flow cytometry. The results are shown in Figure 5.

 As can be seen, the results of fluorescence microscopy showed that the cellular uptake of both targeted and non-targeted bilosomes had occurred; however, a stronger florescence intensity was found for FA-bilosomes at each time point, as compared with the non-targeted ones. The results of the flow cytometry study were also consistent with that obtained from fluorescence microscopy. Based on the geometric means of each histogram in Figure 5c, the mean cellular fluorescence intensity for FA-bilosomes was approximately 1.25 and 2.84 times greater than that in the non-targeted bilosomes and control group, respectively. The results, thus, indicated that the introduction of FA improved the cellular uptake of nanobilosomes via receptor-mediated endocytosis.

###  Cytotoxicity

**Figure 6:**
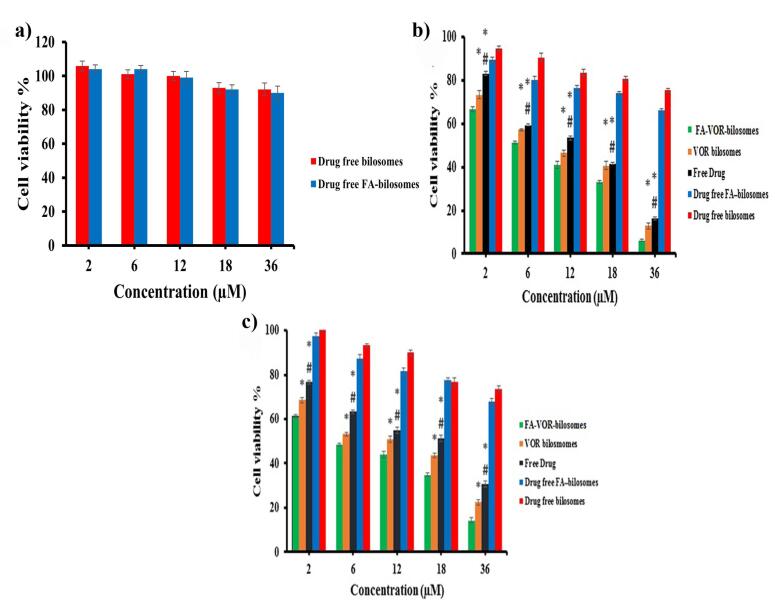
a)* In vitro *cytotoxicity of different formulations on L929 cells* b)* In vitro cytotoxicity of different formulations on MCF-7 cellc) *In vitro *cytotoxicity of different formulations on 4T1 cells* *P* < 0.05 for free drug and VOR- bilosomes vs FA-VOR-bilosomes, # *P* < 0.05 for Free drug vs VOR bilosomes

 The cytotoxicity of targeted and non-targeted drug-free bilosomes against L929 cells is illustrated in Figure 6a. As can be seen, no toxicity was associated with the unloaded drug-free bilosomes, thus indicating the safety of the nanocarriers.

 The cell viability results of the treated MCF_7_ and 4T_1_ cells with free VOR, FA-VOR-bilosomes and VOR-bilosomes are shown in Figure 6b and 6c. It was found that the cytotoxicity of all formulations was dose-dependent, increasing with the concentration of the drug. The results also showed the higher anti-proliferative activity of FA-VOR- bilosomes, as compared with the non-targeted ones, even at very low concentrations (*P* < 0.05). Based on the results, the IC50 value of FA-VOR-bilosomes against MCF-7 and 4T1 cells was 8.64 ± 0.55 µM and 5.75 ± 0.13 µM, respectively; these were both significantly greater than those of VOR- bilosomes, which were 12.42 ± 0.24 µM and 12.25 ± 1.55 µM, respectively (*P* < 0.05). In addition, FA-VOR-bilosomes and VOR-bilosomes were more effective than the free VOR (IC50 for MCF-7: 15.22 ± 0.16 µM and IC50 for 4T1: 18.9 ± 1.41 µM). The present findings are, thus, consistent with the previous research which had shown that drug loaded NPs were more cytotoxic, as compared to the free drug.^39^ The superior antitumor activity of FA-VOR- bilosomes could be due to the high binding affinity of FA ligand attached on the surface of bilosomes to FR overexpressed on the surface of MCF7 and 4T1 cells, which could play an important role in the enhanced receptor mediated internalization of VOR in the cells.^41^ The cytotoxic effect of drug-free FA-targeted bilosomes and non-targeted ones in equal concentrations as the drug-loaded formulation on MCF_7_ and 4T_1_ cells was tested using the MTT assay. Based on data in Figure 6b, 6c, drug-free bilosomes showed a slight dose dependent cytotoxicity, which could be related to the inhibitory effect of LCA on breast cancer proliferation via interfering with multiple anticancer molecular pathways.^10^ Compared with the non-targeted ones, the higher cytotoxicity of drug-free FA-targeted bilosomes (*P* < 0.05) was attributed to the greater intracellular delivery, as discussed before.

## Conclusion

 Our investigation revealed that FA-VOR-bilosomes could be successfully acquired through the thin-film hydration method. The optimal composition of FA-VOR-bilosomes was prepared according to the recommendations of the design expert, which involved 200 mg of lecithin, 80 mg of VOR, 40 mg of LCA, and a sonication time of 4 minutes. The results of the *in vitro* studies indicated that our delivery system did not exhibit any significant toxicity towards the L929 cell line. Furthermore, it was demonstrated that FA-VOR-bilosomes had a higher level of anti-proliferative activity, in comparison to the free VOR, in both MCF-7 and 4T1 cell lines. Additionally, it was observed that the uptake of bilosomes was dependent on the duration of exposure. The findings of our current investigation, thus, suggest that the novel bilosome holds a great potential as a drug delivery system for VOR, serving as a potent chemotherapeutic agent for breast cancer.

## Competing Interests

 The authors report no conﬂicts of interest.

## Ethical Approval

 The study received ethical approval from the Isfahan University of medical sciences research ethics committee, Isfahan, Iran, and reference number IR.MUI.RESEARCH.REC.1400.207.
